# Job Attractiveness and Job Satisfaction of Dental Hygienists: From Japanese Dental Hygienists’ Survey 2019

**DOI:** 10.3390/ijerph18020755

**Published:** 2021-01-17

**Authors:** Yuki Ohara, Yoshiaki Nomura, Yuko Yamamoto, Ayako Okada, Noriyasu Hosoya, Nobuhiro Hanada, Hirohiko Hirano, Noriko Takei

**Affiliations:** 1 Japanese Dental Hygienists’ Association, Tokyo 169-0071, Japan; nori@pm-ms.tepm.jp; 2 Research Team for Promoting Independence and Mental Health, Tokyo Metropolitan Institute of Gerontology, Tokyo 173-0015, Japan; h-hiro@gd5.so-net.ne.jp; 3 Department of Translational Research, Tsurumi University School of Dental Medicine, Yokohama 230-8501, Japan; nomura-y@tsurumi-u.ac.jp (Y.N.); hanada-n@tsurumi-u.ac.jp (N.H.); 4 Department of Endodontology, Tsurumi University School of Dental Medicine, Yokohama 230-8501, Japan; yamamoto-y@tsurumi-u.ac.jp (Y.Y.); hosoya-n@tsurumi-u.ac.jp (N.H.); 5 Department of Operative Dentistry, Tsurumi University School of Dental Medicine, Yokohama 230-8501, Japan; okada-a@tsurumi-u.ac.jp; 6 Department of Dental and Oral Surgery, Tokyo Metropolitan Geriatric Hospital, Tokyo 173-0015, Japan

**Keywords:** dental hygienist, job attractiveness, job satisfaction, work environment

## Abstract

Job attractiveness and job satisfaction are important factors in the continuity of employment among healthcare professionals. The aim of this study was to assess job satisfaction and job attractiveness among dental hygienists in Japan. The Japan Dental Hygienists Association conducted a survey of the employment status of Japanese dental hygienists in 2019. Questionnaires were distributed to all 16,722 members, and 8932 were returned (Collection rate: 53.4%). Data from 7869 currently working dental hygienists were analysed in this study. We analysed seven items of job attractiveness, 14 items of job satisfaction, and 13 items of request for improving the working environment. Item response theory and structural equation modelling (SEM) were utilized for the analysis. For attractiveness of dental hygienists’ work, respondents placed greater emphasis on the fact that dental hygienists needed national qualifications rather than on income stability. SEM showed that job satisfaction consisted of two factors, ‘Value for work’ and ‘Working environment’, as did job attractiveness, with ‘Contribution’ and ‘Assured income’. Value for work affects the contribution to people, and, employment environment affects assured income. Improving job satisfaction and work environments could help to improve the employment rate of dental hygienists, which could positively influence patient care.

## 1. Introduction

The Japanese Dental Hygienists Law states that the mission of dental hygienists is the prevention of oral disease under the instruction of dentists by following treatments, including the mechanical removal of deposits found on the healthy root surface and under healthy free gingiva, drug application on the tooth and oral cavity, assisting in dental treatment, and oral health instructions [1]. Dental hygienists in Japan play an important role as healthcare professionals and have been asked to perform a wide variety of clinical practice skills in the Japanese super-aging society. There is a demand for visiting home dental care, oral care for hospitalised patients, and oral health management for older people requiring long-term care. Previous studies have revealed that oral health management of dental hygienists for older adults or hospitalised patients is effective in improving not only oral health, but also general health conditions [2,3,4], thus, emphasizing the social role of dental hygienists as professionals in oral health management. However, the employment rate of dental hygienists in Japan is very low compared to other countries [5,6]. According to a national survey from 2014, the number of registered dental hygienists in Japan was approximately 250,000, but the number of employed dental hygienists was 116,299 [7]. An insufficient number of dental hygienists may lead to serious problems that affect the supply of dental health care services. Prevention of leaving jobs and support for re-employment are important in maintaining a stable employment rate. Therefore, the development of a positive working environment for dental hygienists is important [7]. 

Job satisfaction is an important prerequisite for a good work environment [8]. Previous studies have reported that job satisfaction is a key factor in continuing employment, especially for healthcare professionals, including dental professionals [9,10,11]. Johns et al. reported that perceived job boredom and lack of benefits helped determine whether a dental hygienist would leave clinical practice. However, salary was implicated as a reason for continuing work [12]. Given these findings, perceived job attractiveness and satisfaction, including employment stability and specialty as a dental hygienist, may lead to motivation and positive attitudes toward work, which in turn may promote individual career formation. 

To ensure stability of the dental hygienist workforce, it is necessary to determine which issues affect dental hygienists and analyse their effects on job attractiveness and satisfaction. However, little is known about how dental hygienists perceive their job attractiveness and satisfaction in Japan. The aim of this study was to clarify the issues of Japanese dental hygienists regarding their job satisfaction, job demands, and work environment.

## 2. Materials and Methods

### 2.1. Study Design and Participants

The Japan Dental Hygienists Association has been conducting surveys on the employment status of dental hygienists in Japan every five years since 1981 [5]. Anonymous questionnaires were distributed to all members of the Japan Dental Hygienists Association on 16 October 2019 by post, and the questionnaires returned by 30 November 2019 were used for the analysis. A total of 16,722 questionnaires were distributed by post, and 8932 were returned (collection rate was 53.4%). Among them, 1063 were from dental hygienists leaving their jobs, which were removed from the analysis, since the data whose did not worked as dental hygienists at the time of the survey, might not reflect the actual situation. This study was approved by the Ethics Committees of the Tsurumi University School of Dental Medicine (approval No. 1837), which was conducted in accordance with the Declaration of Helsinki. Informed written consent was obtained from all participants.

### 2.2. Questionnaire

The questionnaire used in this study consisted of 101 items related to demographic factors, employment status, work content, value of work, etc. We analysed 34 items regarding job attractiveness and satisfaction in addition to the factors dental hygienists feel would improve the work environment. The questionnaires originally created by authors. Job attractiveness was evaluated by seven dichotomous questions about, for example, being a professional, national qualification, and income stability. The questionnaire regarding job satisfaction consisted of 14 items rated on a five-point ordinal scale. The questionnaire regarding the factors dental hygienists feel would improve the work environment consisted of 13 dichotomous questions. 

### 2.3. Statistical Analysis

Cross-tabulation was performed on age group and the items of job attractiveness, and the factors dental hygienists feel would improve the work environment. Correspondence analysis was performed with this cross-tabulation. To visualize the relationships, the results were illustrated graphically as biplots [13]. A three-parameter logistic model with item response theory (IRT) analysis was applied to calculate item discrimination, item difficulties, and item guesses for job attractiveness and satisfaction [1,13,14]. Item response and information curves are graphically illustrated. The analyses were carried out using R software version 3.50 (Institute for Statistics and Mathematics, Wien, Australia) with the LTR and irtoys packages using the following formula:

(1) $$P_{i}\left(\theta \right)=\frac{\left(1-c_{i}\right)}{1+e^{-Da_{i}\left(\theta -b_{i}\right)}}$$

where *a_i_*: discrimination, b*_i_*: difficulty and *c_i_*: guessing.

Factor analysis with varimax rotation was performed to determine the latent variables for structural equation modelling (SEM). The structural relationship between job attractiveness and job satisfaction was calculated using AMOS software (24.0, IBM, Tokyo, Japan).

## 3. Results

### 3.1. Participant Characteristics 

**Figure 1:**
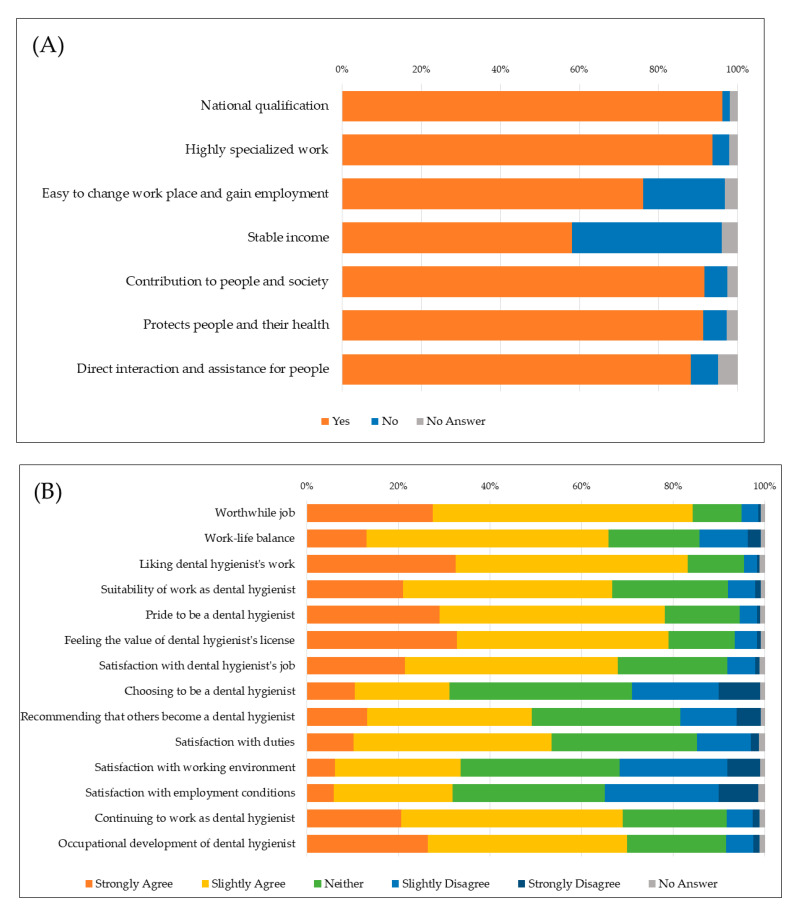

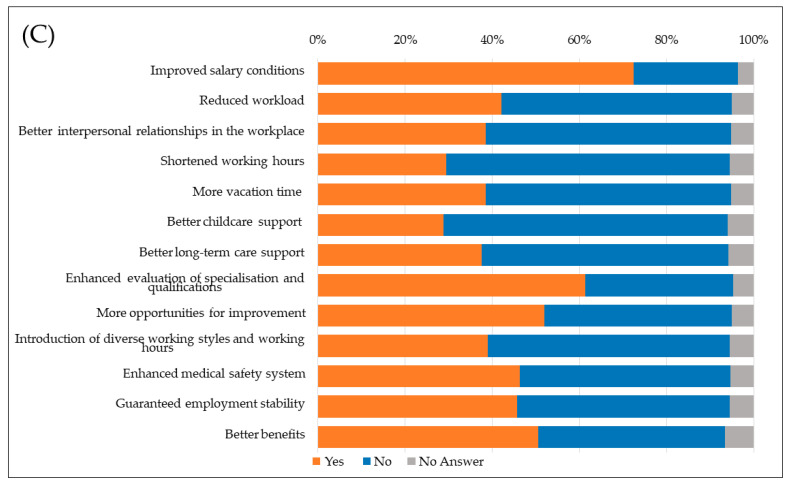
Simple tabulation of participants’ response to each questionnaire. Bar graphs shows the participant’s response to each questionnaire regarding job attractiveness (**A**), job satisfaction(**B**), and the factors dental hygienists feel would improve the work environment (**C**).

The age of the participants was 46.4 ± 11.9 years (median: 48 years, range: 20–81 years). Thirty-five participants (0.4%) were men. The year of experience as a licensed dental hygienist was 20.2 ± 11.4 years (median: 20, range: 0–61). Figure 1 shows the results of descriptive statistics for the items of job attractiveness (A), job satisfaction (B), and the factors dental hygienists feel would improve the work environment (C). In relation to the reason dental hygienist work was attractive, the highest percentage cited ‘National license’ (95.8%), followed by ‘Highly specialised work’ (93.2%) and ‘Contributions to people and society’ (91.3%). For job satisfaction, the highest proportion cited ‘Worthwhile job’ (84.3%), followed by ‘Liking dental hygienists’ work’ (83.2%), and ‘Feeling the value of hygienist’s license’ (79.0%). Regarding the factors dental hygienists feel would improve the work environment, the most frequently responses were ‘Improved salary’ (72.5%), followed by ‘Enhanced evaluation of specialisation and qualification’ (61.3%). Biplots of age group for each question are presented in Figure S1. 

### 3.2. IRT Analysis for Job Attractiveness and the Factors Dental Hygienists Feel Would Improve the Work Environment 

Using factor analysis we categorised the 14 items regarding job satisfaction into two factors: ‘Value for work’ and ‘Working environment’. Similarly, the seven items of job attractiveness were categorised into two factors, ‘Contribution’ and ‘Assured income’. The 13 items regarding the factors dental hygienists feel would improve the work environment were categorised into three factors (Table S1). The attractiveness of dental hygienists’ work and the aforementioned factors were analysed using a 3 three-parameter logistic model based on IRT. 

**Figure 2:**
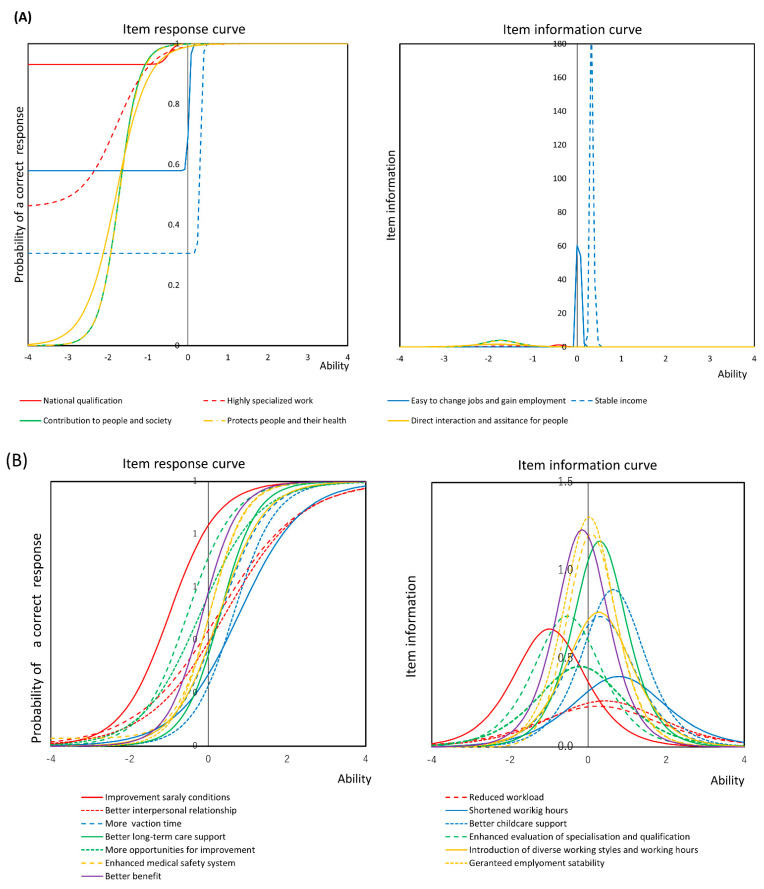
Item response curve and item information curve for the items regarding job attractiveness of dental hygienists’ work and the factors dental hygienists feel would improve the work environment. (**A**) Job attractiveness (**B**) The factors dental hygienists feel would improve the work environment.

Figure 2 shows item response curves and item information curves for the attractiveness of dental hygienists’ work (A) and the factors dental hygienists feel would improve the work environment (B). The constructed models are shown in Table S2. For attractiveness of dental hygienists’ work, item response curves shifted backward. The steepness of the curve at its inflexion point provides a measure of the discriminatory power of the item. Discrimination refers to how well an item can distinguish between respondents with low ability levels and those with high ability levels. In this case, respondents with high ability indicates responded ‘Yes’ often for the items, whereas respondents with low ability levels a low are relatively flat have low discrimination.

The horizontal axis shows the participant’s ability and the item response curve axis shows the positive response to each item. Ability, shown on the horizontal axis, indicates the standardized weighted sum of the positive response of the items. That is, the closer the forward area, the more negative the question, and the closer the backward area, the more likely the answer is positive. The item response curve shows how precisely each item measures latent traits at various levels. A greater area under this curve indicates that ‘Yes’ was answered for all items at a higher rate, and these items may shape attractiveness to work for dental hygienists. Among them, items of ‘National qualification’ and ‘Easy to change work place and gain employment’ had a probability of higher than 0.5 at the origin point, which indicates that more than half of dental hygienists answered ‘Yes’ for these items. The item response curves for ‘Stable income’ and ‘Easy to change work place and gain employment’ were steep, which indicates that the responses to these items have a clear cut-off point. The three items of ‘Protects people and their health’, ‘Direct interaction and assistance for people’, and ‘Contribution to people and society’ were located backward direction, which indicates that most of the dental hygienists answered ‘Yes ‘for these items. Regarding the item information curve of job attractiveness, the item ‘National qualification’ had little information and was in a backward direction, which indicates that most dental hygienists responded ‘Yes’ to these items. ‘Stable income’ had the highest item information, followed by ‘Easy to change work place and gain employment’. These curves are located near the Y axis, which indicates that about half of the dental hygienists responded ‘Yes’ to these items. Where dental hygienists answered ‘Yes’ to these items, they responded ‘Yes’ to all other items.

Figure 2B shows the item response and information curves for the working environment. Many participants indicated a need to improve salary conditions, such as having regular pay raises and enhancing evaluation of specialisation and qualification. Neither IRT, nor item information curves regarding the working environment were as systematic as attractiveness to work, and there was no characteristic trend. Item response curves and item information curves for job attractiveness and demands for professional improvement analysed per factor extracted by factor analysis are shown in Figure S2. All items on the item response curve for job attractiveness were shifted in a backward direction; thus, many dental hygienists considered all items to be important, and in the item information curve, peaks for ‘Easy to change work place and gain employment’ indicated a tendency for dental hygienists to preferentially place importance relative to other items. In relation to the factors dental hygienists feel would improve the work environment, the tendency of the item response curve showed that the proportion of dental hygienists who answered ‘Improved salary conditions’ was relatively high, and items such as ‘Better long-term care support’ were less emphasised. Information from item information curves indicated that the items of guaranteed employment stability had greater information.

### 3.3. SEM for Job Satisfaction and Attractiveness 

**Figure 3:**
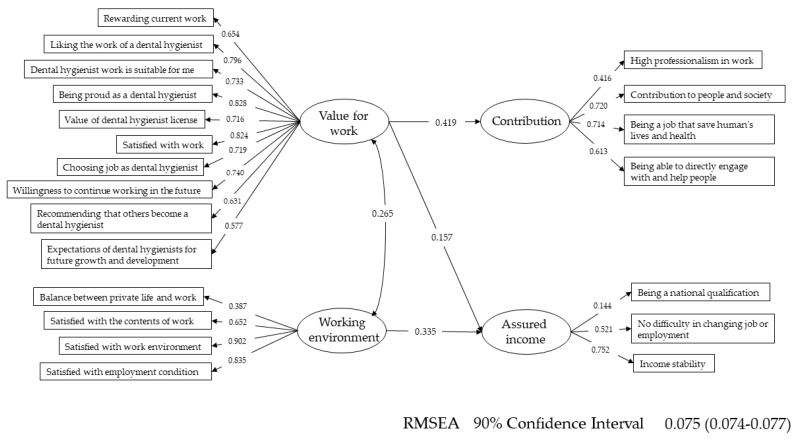
Path diagram of job satisfaction and attractiveness of dental hygienists’ work; RMSEA: Root Mean Square Error of Approximation.

SEM was conducted to visualise the influence of job satisfaction on the attractiveness of dental hygienists’ jobs (Figure 3). All paths were statistically significant. ‘Value for work’ significantly affected ‘Contribution’, and ‘Working environment’ affected ‘Assured income’ to some extent. 

## 4. Discussion

In this large-scale study of dental hygienists in Japan, we investigated the association between job attractiveness and satisfaction, and the current status of the factors dental hygienists feel would improve the work environment. To the best of our knowledge, this is the first report describing the detailed characteristics of occupational awareness among Japanese dental hygienists, which cannot be clarified by the results of simple descriptive statistics. This study has been conducted by the Japan Dental Hygienists Association every five years. Many of the items were dichotomous responses. This survey confirmed the results of the previous survey. Dichotomous responses lack depth of information compared to those rated on a Likert-type scale. However, when applied to item response analysis, results obtained using dichotomous variable are easy to interpret [15]; this study utilized the merits of such variables. Item response theory analysis is a powerful analytical method, especially for dichotomous variables. It is widely used in educational research and tests, such as the widely-known TOEFL. It is also applicable in medical research. Valuable information, rather than a simple descriptive analysis of frequency, can be presented using IRT. The slope and location of item information curve can provide valuable information on the response pattern in a questionnaire. We have been frequently applying IRT for in our research studies. When interpreting the descriptive analysis of job attractiveness, more than 90% of dental hygienists gave positive responses for all items except ‘Stable income’ and ‘Easy to change work place and gain employment’. Moreover, the item response curve revealed that the curves of all items were shifted backward, that is, many respondents responded that the work of dental hygienists was attractive. This result suggests that most dental hygienists find value in their jobs. Therefore, the strength that the dental hygienists perceive attractiveness of these tasks is an important factor for their work continuity. Most dental hygienists recognised attractiveness in the stability of their status as a worker, that is, having a national qualification made it easy to change where they work. 

Notably, direct involvement with people and contributions to life and society tended to be perceived as attractive only if other factors were met. According to the item information curve, income stability and easy to change work place and gain employment had high item information. These two items were more attractive than the other items. The results of IRT and factor analysis indicated that many dental hygienists considered that easy to change work place and stable income were more important than national qualifications (Table S1[A]). In contrast, for ‘Contribution’ factors, all items were presented as sigmoidal curves. This suggests that dental hygienists find more value regarding aspects related to the contribution of their work as job attractiveness increases (Table S1B). 

Factors directly linked to daily life, such as employment status and income stability, may be prerequisites for the attractiveness of work as a dental hygienist. With respect to t the factors dental hygienists feel would improve the work environment, item information curves of salary and appraisal of specialty and license were backwards. This indicates that many dental hygienists requested these two items rather than other working conditions. Conversely, item information curves for childcare support and shortened working hours were forward-facing, indicating that a limited number of dental hygienists requested for the improvement of these two conditions. When comparing the item response curve and item information curves of the factors dental hygienists feel would improve the work environment with attractiveness, curves were gentle sloped sigmoid curves and were in a limited area. This indicates that even though the salary and appraisal of specialty were common requests, the need to improve other conditions depended about each dental hygienist. In other words, the perception of the working environment may be influenced by the circumstances and view of each dental hygienist; thus, a subdivided validation of each of these factors is necessary. 

A previous study reported that reducing the workload, enhancing welfare, and career developments were associated with job satisfaction among healthcare staff in China [16]. However, the results of this study showed that the demands about salary and employment stability were more pronounced than the workload. This trend of salary emphasis was like findings from previous studies about dental care providers [17,18,19]. 

The results from the SEM showed that factors related to the working environment significantly influenced factors of assured income regarding job attractiveness. Previous studies have also reported that turnover of healthcare professionals is caused by dissatisfaction with their work, but it is inferred that the factors causing dissatisfaction may differ depending on job content and educational background [16,20]. In particular, improvement in salary may improve the job satisfaction of dental hygienists in Japan. Detailed verification is necessary for the improvement of working conditions of dental hygienists for the planning of specific measures to prevent turnover. Therefore, further study is necessary to investigate the association between leaving jobs and job satisfaction. The results of the SEM showed that the job satisfaction of dental hygienists presented their characteristics as professionals. Supporting people’s health, such as contributions to people and society, had high loadings. Ayers et al. reported that one of the independent factors associated with career satisfaction among New Zealand dental therapists was whether they felt that they were a valued part of the dental community [19], so increasing the value of work may increase job satisfaction. The improvement of both the contribution to people and society and assured salary may be issues for ensuring dental hygienists’ satisfaction and improving the quality of dental services in Japan.

There are some limitations to the present study. First, the participants may have a variety of backgrounds. For example, years of education before obtaining a dental hygienist’s license, years of clinical experience, and place of employment may have led to differences in job attractiveness, satisfaction, and the factors dental hygienists feel would improve the work environment. Correspondence analysis also revealed the characteristics of the participants according to their generations, which warrants the need for in-depth examination in the future [21,22]. Second, the duties of dental hygienists are stipulated by the legislation and regulations of each country, and the specific content varies widely, so the results have limited generalizability outside of Japan. Job satisfaction is a key factor in the stable career formation of healthcare providers; therefore, studies comparing and examining differences on a global scale are desirable in the future.

## 5. Conclusions

In conclusion, the results indicated that Japanese dental hygienists find that the stability of their occupation and employment is equally important to their contribution to people and society, and that these factors are highly relevant to job satisfaction. Improving job satisfaction and work environments could help prevent high turnover among dental hygienists. In particular, it is important to improve their working environment, so that it leads to improved salary conditions, and enhanced assessment of professionalism and qualifications.

## Data Availability

The data of the present study were used under license for the current study and, therefore, are not publicly available.

## Supplementary Materials

The following are available online at https://www.mdpi.com/1660-4601/18/2/755/s1, Table S1: Results of factor analysis of job satisfaction, job attractiveness, and the factors dental hygienists feel would improve the work environment, Table S2: Three parameter logistic model based on item response theory, Figure S1: Biplots of age group and job attractiveness(A), the factors dental hygienists feel would improve the work environment (B). Navy plots correspond to age group of the participants. Closely located plots are meaning highly coincident, Figure S2: Item response curve and item information curve of for the items regarding job attractiveness of dental hygienists’ work and the factors dental hygienists feel would improve the work environment by each factor.
