# Supplementary material for: Job Attractiveness and Job Satisfaction of Dental Hygienists: From Japanese Dental Hygienists’ Survey 2019
Source: Int J Environ Res Public Health. 2021 Jan 17;18(2):755. doi: 10.3390/ijerph18020755 (PMC7830899; doi:10.3390/ijerph18020755)
Supplement: Supplementary file 1 [file ijerph-18-00755-s001.zip › Supplymental revised20210113.docx]

**Table S1.** Results of factor analysis of job satisfaction, job attractiveness, and the factors dental hygienists feel would improve the work environment

(A) Job satisfaction.

|  | Factor | |
| --- | --- | --- |
|  | 1 | 2 |
| Pride to be a dental hygienist | 0.837 |  |
| Liking dental hygienist work | 0.821 |  |
| Suitability of work as dental hygienist | 0.759 |  |
| Satisfaction with dental hygienist's job | 0.743 |  |
| Continuing to work as dental hygienist | 0.657 |  |
| Feeling the value of dental hygienist's license | 0.652 |  |
| Choosing to be a dental hygienist | 0.639 |  |
| Do you feel that your current work is rewarding? | 0.613 |  |
| Recommending dental hygienist to others | 0.513 |  |
| Occupational development of dental hygienist | 0.482 |  |
| Satisfaction with working environment |  | 0.868 |
| Satisfaction with employment conditions |  | 0.855 |
| Satisfaction with duties |  | 0.559 |
| Work-life balance |  | 0.315 |
| Total | 4.971 | 2.690 |
| % of variance | 35.505 | 19.215 |
| Cumulative (%) | 35.505 | 54.721 |

**Table S2.** Three parameter logistic model based on item response theory

(A-1) All items concerning attractiveness of dental hygienist work

|  | Discrimination | Difficulty | Guessing |
| --- | --- | --- | --- |
| National qualification | 11.24815 | -0.48676 | 0.9311451 |
| Highly specialized work | 2.262627 | -1.78691 | 0.4597 |
| Easy to change work place and gain employment | 42.99335 | 0.022919 | 0.5790528 |
| Stable income | 38.34428 | 0.316755 | 0.3057423 |
| Contribution to people and society | 3.97643 | -1.72411 | 0.000265951 |
| Protects people and their health | 3.896564 | -1.71725 | 0.000058663 |
| Direct interaction and assistance for people | 2.554057 | -1.79737 | 0.000159643 |

(A-2) Factor 1

|  | Discrimination | Difficulty | Guessing |
| --- | --- | --- | --- |
| National qualification | 1.329884 | -3.5052887 | 0.05073262 |
| Easy to change work place and gain employment | 9.887276 | -0.4784935 | 0.27916676 |
| Stable income | 17.921063 | 0.1970955 | 0.23876962 |

(A-3) Factor 2

|  | Discrimination | Difficulty | Guessing |
| --- | --- | --- | --- |
| Highly specialized work | 1.735903 | -2.466865 | 0 |
| Contribution to people and society | 4.023061 | -1.715703 | 0 |
| Protects people and their health | 3.928933 | -1.709512 | 0 |
| Direct interaction and assistance for people | 2.67655 | -1.764223 | 0 |

(B-1) All items for the factors dental hygienists feel would improve the work environment

|  | Discrimination | Difficulty | Guessing |
| --- | --- | --- | --- |
| Improvement salary conditions | 1.6361651 | -0.98629317 | 1.24×10^-14^ |
| Reduced workload | 0.9612751 | 0.26576254 | 1.21×10^-5^ |
| Better interpersonal relationship | 1.0217652 | 0.43171526 | 8.64×10^-6^ |
| Shortened working hours | 1.2642544 | 0.78717276 | 3.08×10^-4^ |
| More vacation time | 1.7204456 | 0.31368487 | 8.04×10^-7^ |
| Better childcare support | 1.890449 | 0.64719364 | 4.59×10^-6^ |
| Better long-term care support | 2.1624933 | 0.3001754 | 1.10×10^-5^ |
| Enhanced evaluation of specialization and qualification | 1.7232079 | -0.52560727 | 1.24×10^-11^ |
| More opportunities for improvement | 1.3511519 | -0.20591574 | 3.06×10^-7^ |
| Introduction of diverse working styles and working hours | 1.748466 | 0.28239159 | 2.13×10^-7^ |
| Enhanced medical safety system | 2.2644922 | 0.06077934 | 2.98×10^-2^ |
| Guaranteed employment stability | 2.2858315 | 0.03108183 | 5.12×10^-8^ |
| Better benefit | 2.2207998 | -0.14681951 | 1.35×10^-7^ |

(B-2) Factor 1

|  | Discrimination | Difficulty | Guessing |
| --- | --- | --- | --- |
| Improvement salary conditions | 1.6048756 | -0.998478 | 2.74×10^-7^ |
| Better interpersonal relationship | 0.9977948 | 0.4449923 | 3.05×10^-4^ |
| Shortened working hours | 1.3366598 | 0.8668083 | 3.06×10^-2^ |
| More vacation time | 1.8250407 | 0.3140985 | 3.09×10^-4^ |
| Better long-term care support | 2.6360759 | 0.6288968 | 2.07×10^-2^ |
| Better childcare support | 2.9728431 | 0.3199872 | 2.17×10^-2^ |
| Enhanced evaluation of specialization and qualification | 1.6927186 | -0.5284169 | 4.08×10^-7^ |
| More opportunities for improvement | 1.2159183 | -0.2148183 | 2.34×10^-5^ |
| Better benefit | 1.6885186 | -0.1581892 | 6.27×10^-6^ |

(B-3) Factor 2

|  | Discrimination | Difficulty | Guessing |
| --- | --- | --- | --- |
| Reduced workload | 0.6049744 | 0.4040813 | 0.000502055 |
| Introduction of diverse working styles and working hours | 1.4024687 | 0.3353458 | 0.000108195 |
| Enhanced medical safety system | 3.7943081 | 0.1633017 | 0.08966136 |
| Guaranteed employment stability | 4.3859244 | 0.1732813 | 0.083888363 |

(B) Job attractiveness

|  | Factor | |
| --- | --- | --- |
|  | 1 | 2 |
| Contribution to people and society | 0.725 |  |
| Protects people and their health | 0.712 |  |
| Direct interaction and assistance for people | 0.605 |  |
| Highly specialized work | 0.376 |  |
| Easy to change jobs and gain employment |  | 0.722 |
| Stable income |  | 0.538 |
| National qualification |  | 0.170 |
| Total | 1.557 | 0.904 |
| % of variance | 22.242 | 12.919 |
| Cumulative (%) | 22.242 | 35.161 |

(C) The factors dental hygienists feel would improve the work environment

|  | Factor | | |
| --- | --- | --- | --- |
|  | 1 | 2 | 3 |
| Guaranteed employment stability | 0.701 |  |  |
| Better benefit | 0.666 |  |  |
| Enhanced medical safety system | 0.663 |  |  |
| Enhanced evaluation of specialization and qualification | 0.495 |  |  |
| More opportunities for improvement | 0.491 |  |  |
| Improvement salary conditions | 0.423 |  |  |
| Introduction of diverse working styles and working hours | 0.366 |  |  |
| Shortened working hours |  | 0.707 |  |
| Reduced workload |  | 0.644 |  |
| More vacation time |  | 0.421 |  |
| Working relationship |  | 0.334 |  |
| Better interpersonal relationship |  |  | 0.794 |
| Better long-term care support |  |  | 0.537 |
| Total | 2.505 | 1.552 | 1.293 |
| % of variance | 19.267 | 11.938 | 9.945 |
| Cumulative (%) | 19.267 | 31.206 | 41.151 |

**
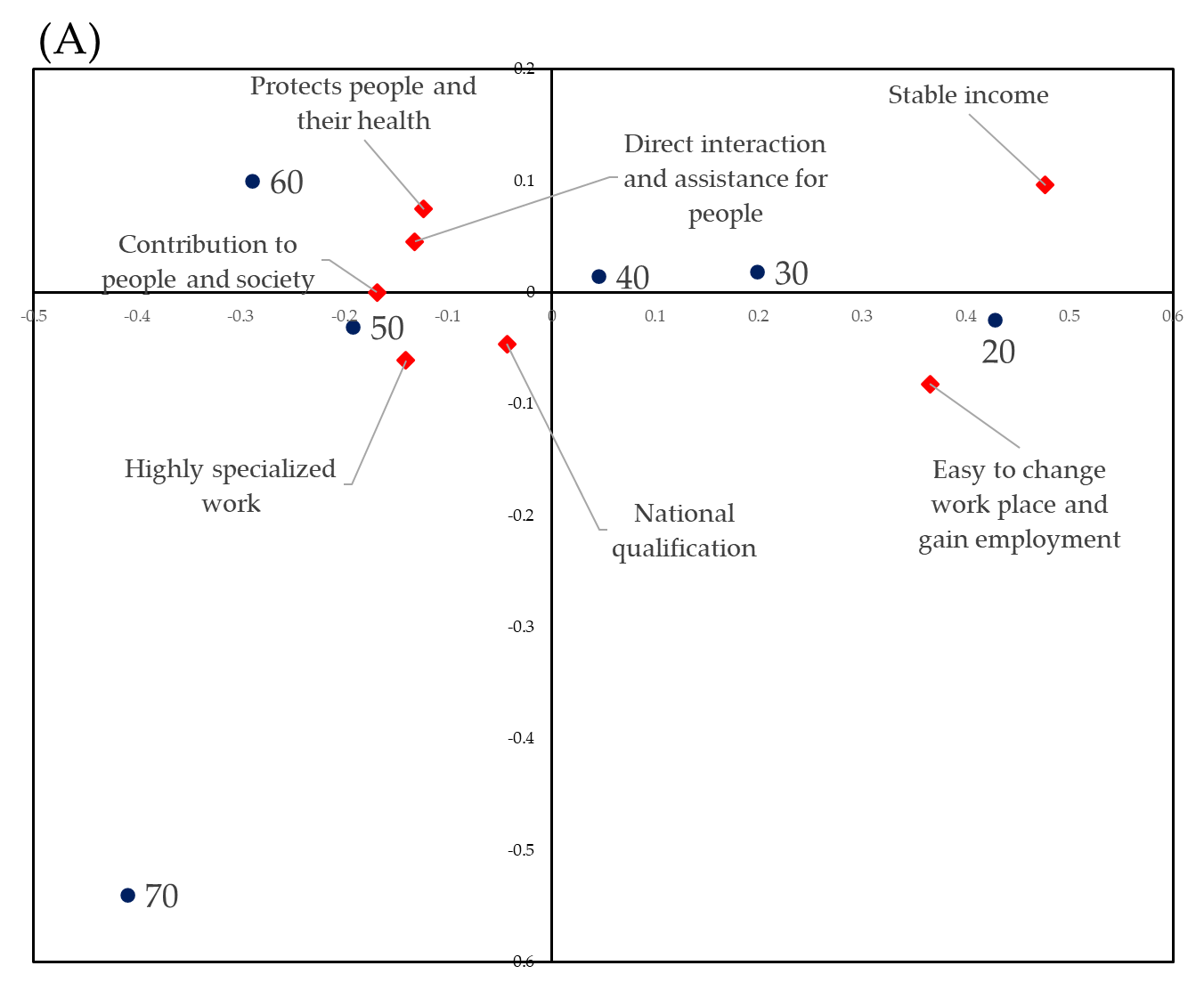
**

**
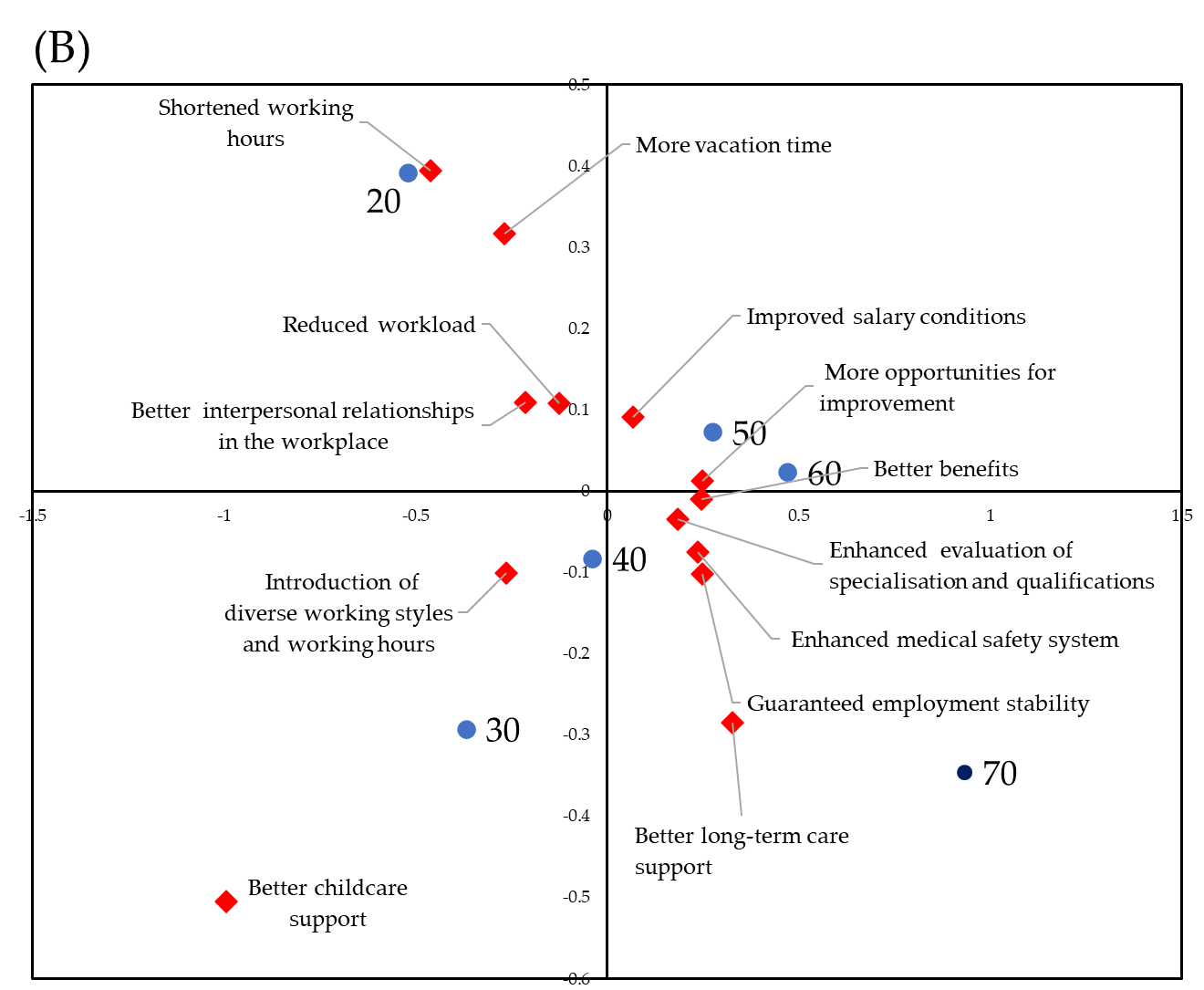
**

**Figure S1.** Biplots of age group and job attractiveness(A), the factors dental hygienists feel would improve the work environment (B). Navy plots correspond to age group of the participants. Closely located plots are meaning highly coincident.


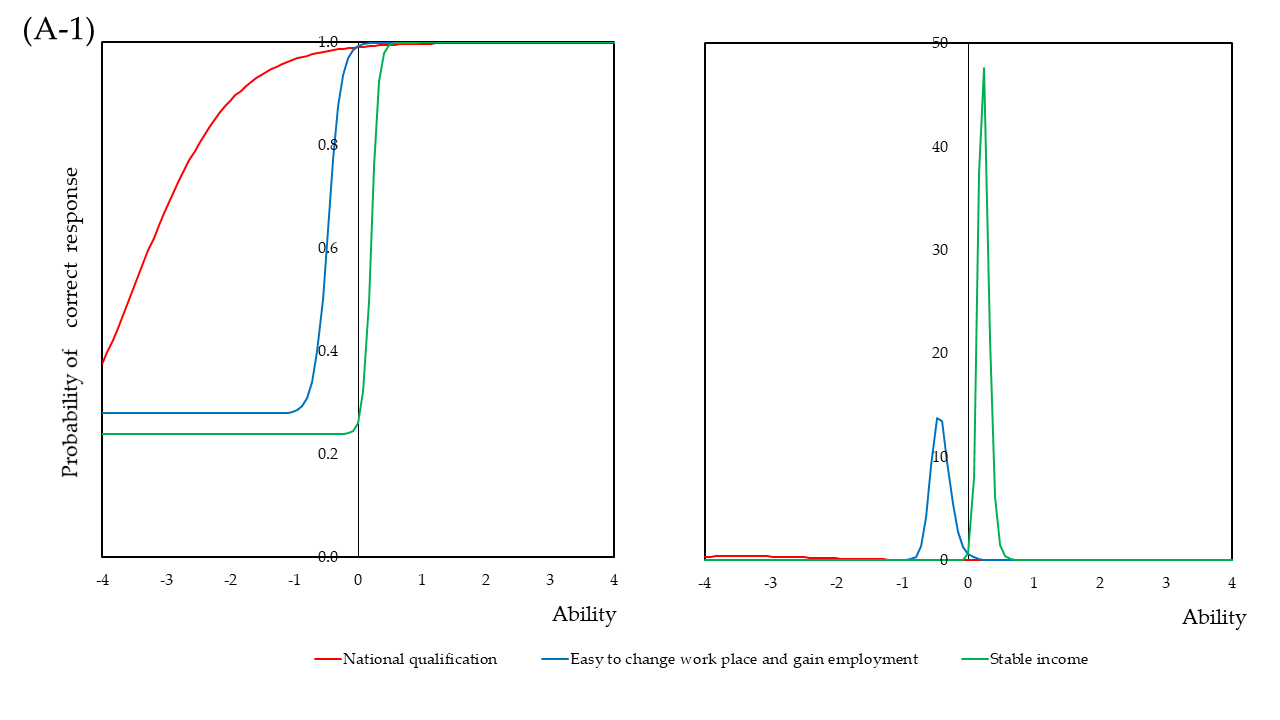


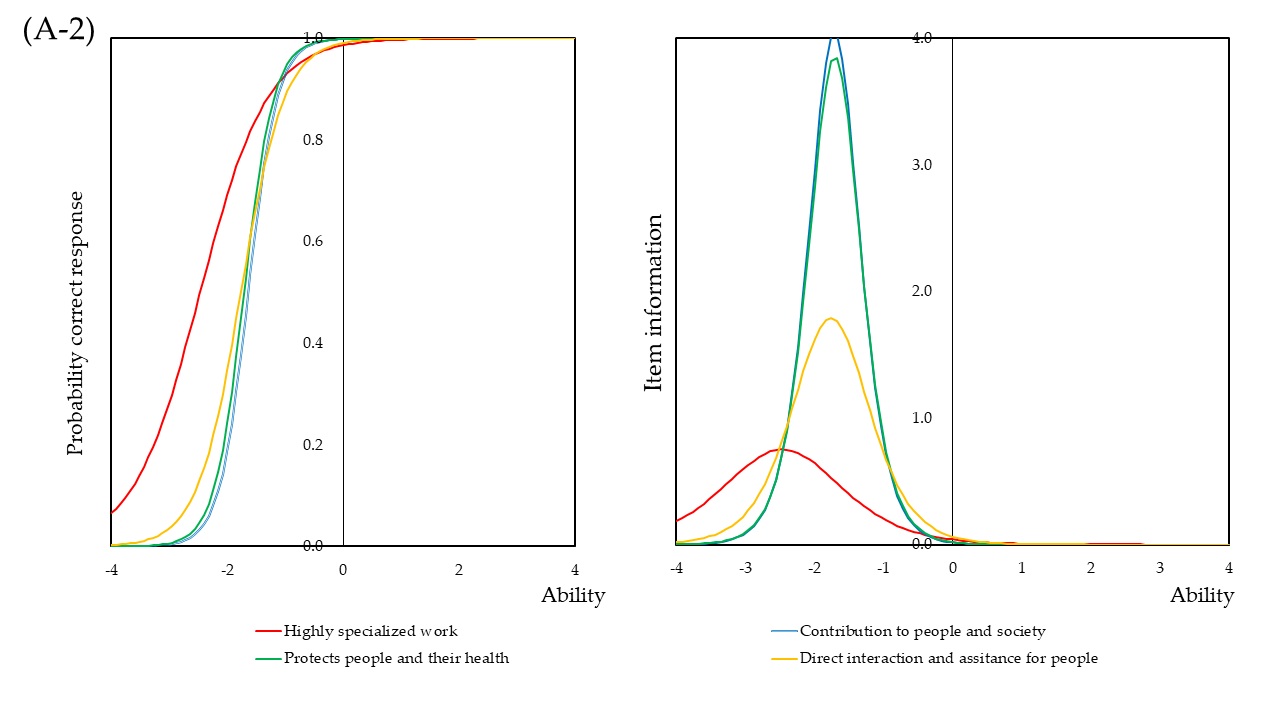


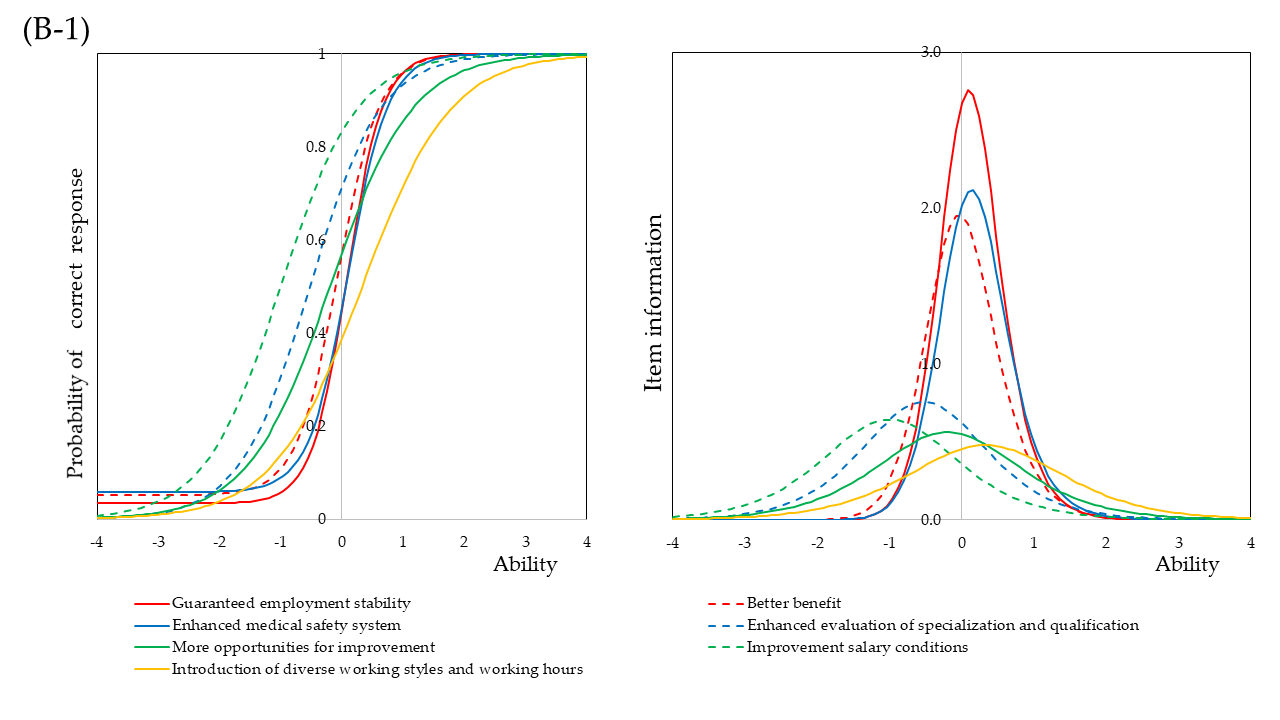

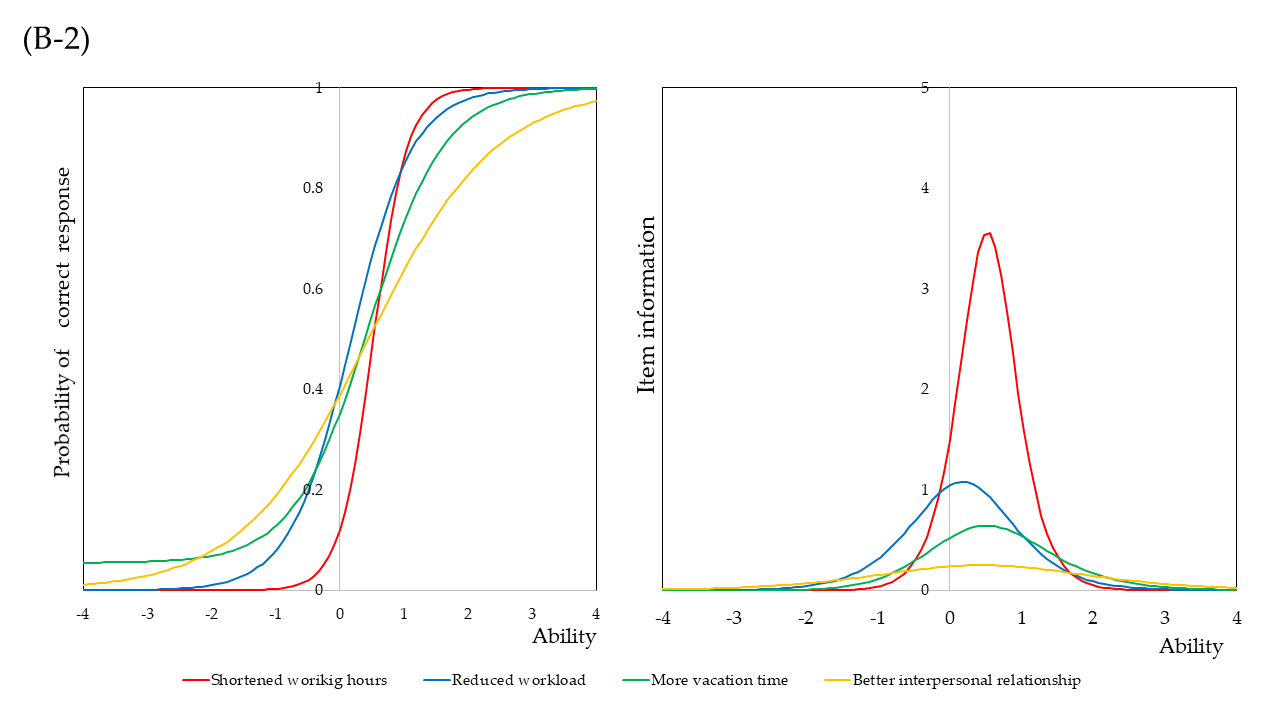


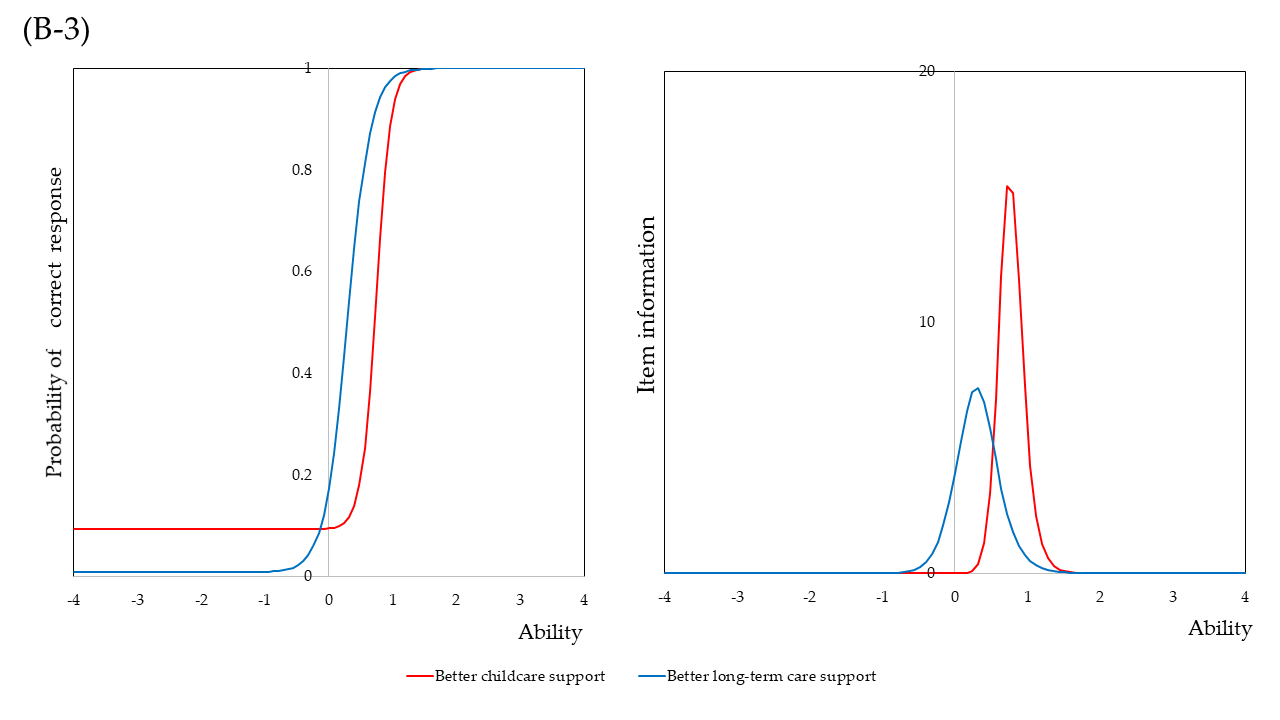


**Figure S2.** Item response curve and item information curve of for the items regarding job attractiveness of dental hygienists’ work and the request for working environment by each factor: Job attractiveness (A), the factors dental hygienists feel would improve the work environment (B)
