# Supplementary material for: Job Attractiveness and Job Satisfaction of Dental Hygienists: From Japanese Dental Hygienists’ Survey 2019
Source: Int J Environ Res Public Health. 2021 Jan 17;18(2):755. doi: 10.3390/ijerph18020755 (PMC7830899; doi:10.3390/ijerph18020755)
Supplement: Supplementary file 1 [file ijerph-18-00755-s001.zip › Supplymental revised20210113.pdf]

Table S1. Results of factor analysis of job satisfaction, job attractiveness, and the factors dental hygienists feel would improve the work environment

(A) Job satisfaction.

	Factor	
	1	2
Pride to be a dental hygienist	0.837	
Liking dental hygienist work	0.821	
Suitability of work as dental hygienist	0.759	
Satisfaction with dental hygienist's job	0.743	
Continuing to work as dental hygienist	0.657	
Feeling the value of dental hygienist's license	0.652	
Choosing to be a dental hygienist	0.639	
Do you feel that your current work is rewarding?	0.613	
Recommending dental hygienist to others	0.513	
Occupational development of dental hygienist	0.482	
Satisfaction with working environment		0.868
Satisfaction with employment conditions		0.855
Satisfaction with duties		0.559
Work-life balance		0.315
Total	4.971	2.690
% of variance	35.505	19.215
Cumulative (%)	35.505	54.721

Table S2. Three parameter logistic model based on item response theory

(A-1) All items concerning attractiveness of dental hygienist work

	Discrimination	Difficulty	Guessing
National qualification	11.24815	-0.48676	0.9311451
Highly specialized work	2.262627	-1.78691	0.4597
Easy to change work place and gain employment	42.99335	0.022919	0.5790528
Stable income	38.34428	0.316755	0.3057423
Contribution to people and society	3.97643	-1.72411	0.000265951
Protects people and their health	3.896564	-1.71725	0.000058663
Direct interaction and assistance for people	2.554057	-1.79737	0.000159643

(A-2) Factor 1

	Discrimination	Difficulty	Guessing
National qualification	1.329884	-3.5052887	0.05073262
Easy to change work place and gain employment	9.887276	-0.4784935	0.27916676
Stable income	17.921063	0.1970955	0.23876962

(A-3) Factor 2

	Discrimination	Difficulty	Guessing
Highly specialized work	1.735903	-2.466865	0
Contribution to people and society	4.023061	-1.715703	0
Protects people and their health	3.928933	-1.709512	0
Direct interaction and assistance for people	2.67655	-1.764223	0

(B-1) All items for the factors dental hygienists feel would improve the work environment

	Discrimination	Difficulty	Guessing
Improvement salary conditions	1.6361651	-0.98629317	1.24×10^{-14}
Reduced workload	0.9612751	0.26576254	1.21×10^{-5}
Better interpersonal relationship	1.0217652	0.43171526	8.64×10^{-6}
Shortened working hours	1.2642544	0.78717276	3.08×10^{-4}
More vacation time	1.7204456	0.31368487	8.04×10^{-7}
Better childcare support	1.890449	0.64719364	4.59×10^{-6}
Better long-term care support	2.1624933	0.3001754	1.10×10^{-5}
Enhanced evaluation of specialization and qualification	1.7232079	-0.52560727	1.24×10^{-11}

More opportunities for improvement	1.3511519	-0.20591574	3.06×10^{-7}
Introduction of diverse working styles and working hours	1.748466	0.28239159	2.13×10^{-7}
Enhanced medical safety system	2.2644922	0.06077934	2.98×10^{-2}
Guaranteed employment stability	2.2858315	0.03108183	5.12×10^{-8}
Better benefit	2.2207998	-0.14681951	1.35×10^{-7}

(B-2) Factor 1

	Discrimination	Difficulty	Guessing
Improvement salary conditions	1.6048756	-0.998478	2.74×10^{-7}
Better interpersonal relationship	0.9977948	0.4449923	3.05×10^{-4}
Shortened working hours	1.3366598	0.8668083	3.06×10^{-2}
More vacation time	1.8250407	0.3140985	3.09×10^{-4}
Better long-term care support	2.6360759	0.6288968	2.07×10^{-2}
Better childcare support	2.9728431	0.3199872	2.17×10^{-2}
Enhanced evaluation of specialization and qualification	1.6927186	-0.5284169	4.08×10^{-7}
More opportunities for improvement	1.2159183	-0.2148183	2.34×10^{-5}
Better benefit	1.6885186	-0.1581892	6.27×10^{-6}

(B-3) Factor 2

	Discrimination	Difficulty	Guessing
Reduced workload	0.6049744	0.4040813	0.000502055
Introduction of diverse working styles and working hours	1.4024687	0.3353458	0.000108195
Enhanced medical safety system	3.7943081	0.1633017	0.08966136
Guaranteed employment stability	4.3859244	0.1732813	0.083888363

(B) Job attractiveness

	Factor	
	1	2
Contribution to people and society	0.725	
Protects people and their health	0.712	
Direct interaction and assistance for people	0.605	
Highly specialized work	0.376	
Easy to change jobs and gain employment		0.722

Stable income		0.538
National qualification		0.170
Total	1.557	0.904
% of variance	22.242	12.919
Cumulative (%)	22.242	35.161

(C) The factors dental hygienists feel would improve the work environment

	Factor		
	1	2	3
Guaranteed employment stability	0.701		
Better benefit	0.666		
Enhanced medical safety system	0.663		
Enhanced evaluation of specialization and qualification	0.495		
More opportunities for improvement	0.491		
Improvement salary conditions	0.423		
Introduction of diverse working styles and working hours	0.366		
Shortened working hours		0.707	
Reduced workload		0.644	
More vacation time		0.421	
Working relationship		0.334	
Better interpersonal relationship			0.794
Better long-term care support			0.537
Total	2.505	1.552	1.293
% of variance	19.267	11.938	9.945
Cumulative (%)	19.267	31.206	41.151

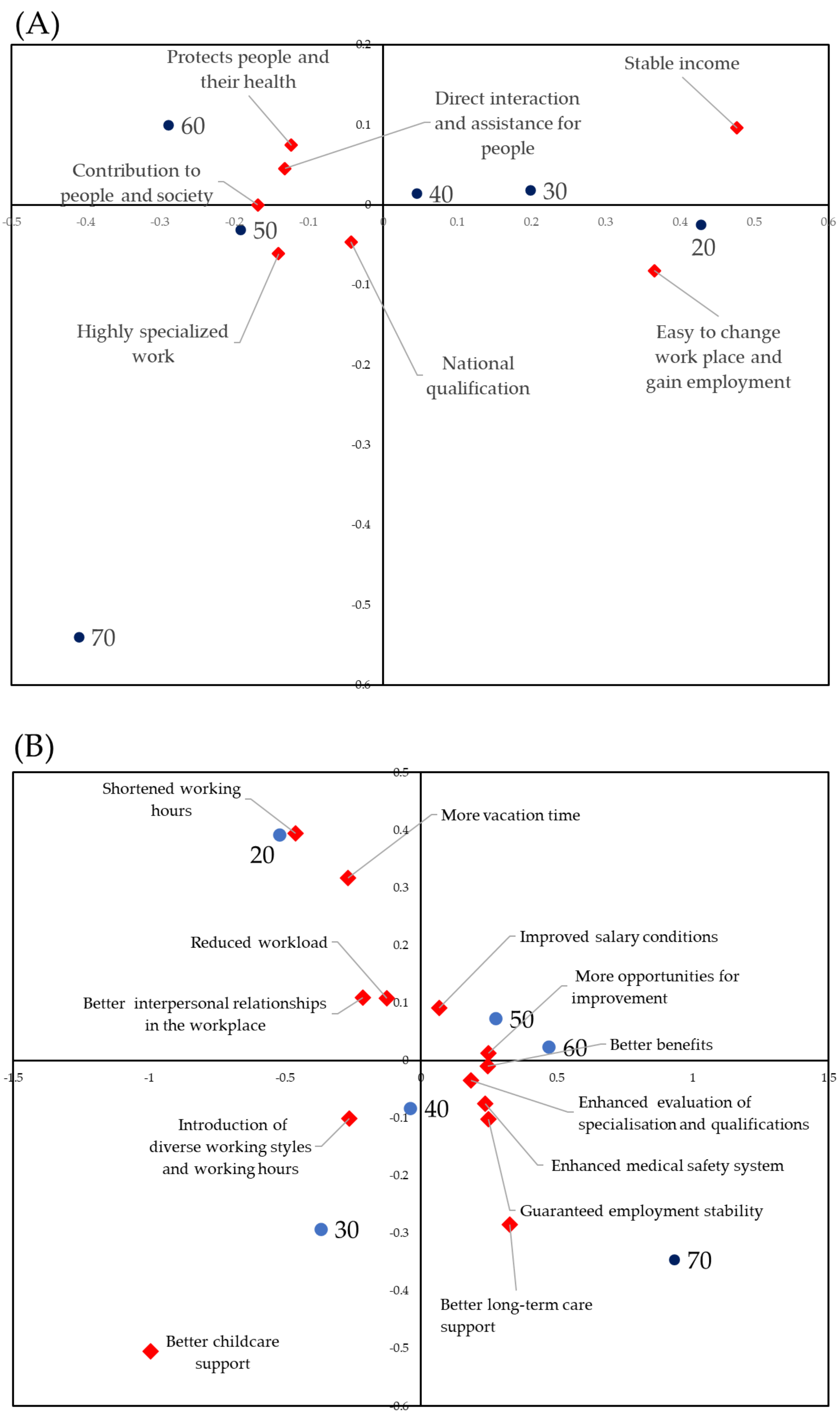

Figure S1. Biplots of age group and job attractiveness(A), the factors dental hygienists feel would improve the work environment (B). Navy plots correspond to age group of the participants. Closely located plots are meaning highly coincident.

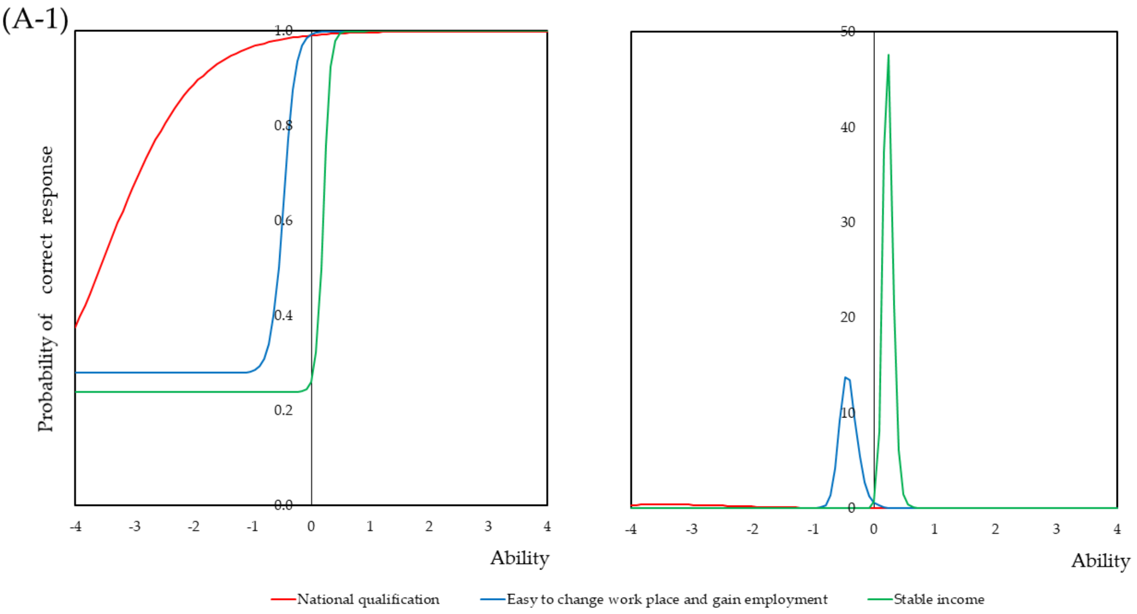
(A-1)
Probability of correct response
Ability
Ability
National qualification
Easy to change work place and gain employment
Stable income

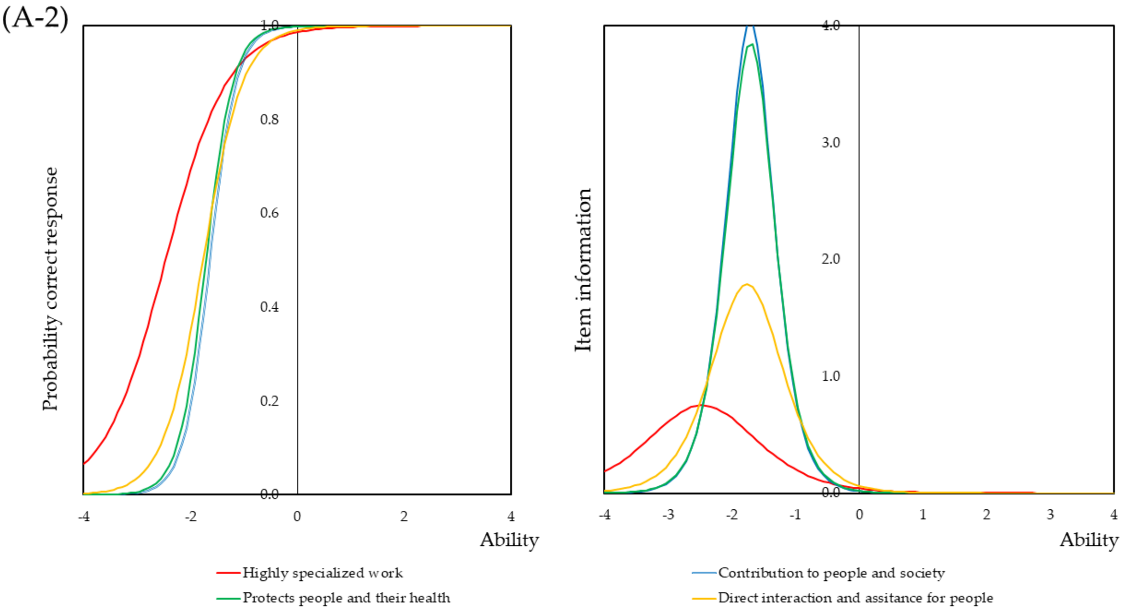
(A-2)
Probability correct response
Item information
Ability
Ability
Highly specialized work
Protects people and their health
Contribution to people and society
Direct interaction and assitance for people

(B-1)

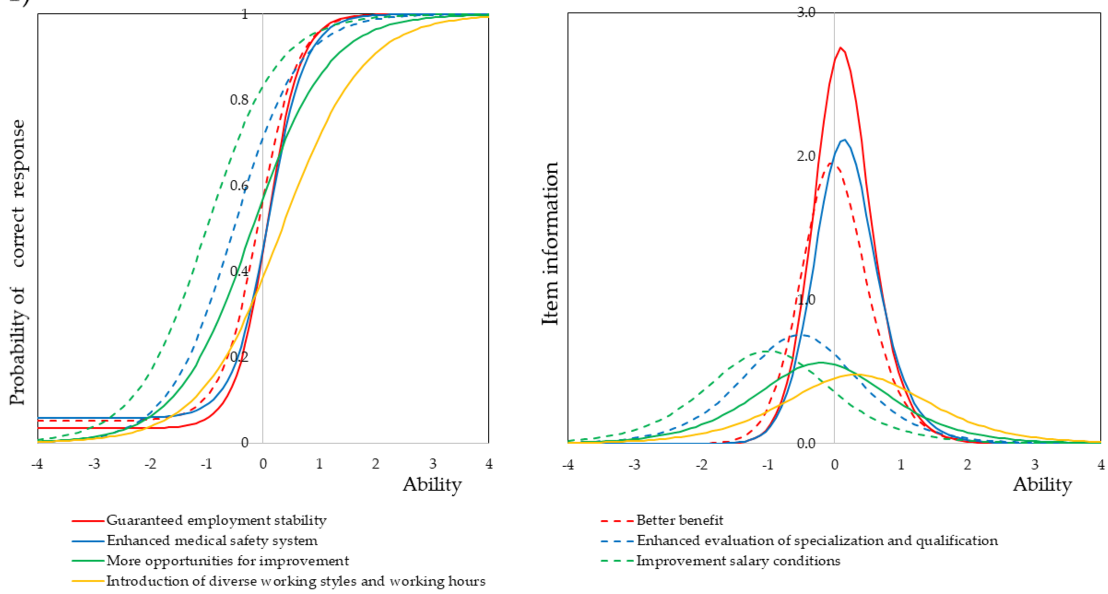
Probability of correct response
Ability
Item information
Ability
Guaranteed employment stability
Enhanced medical safety system
More opportunities for improvement
Introduction of diverse working styles and working hours
Better benefit
Enhanced evaluation of specialization and qualification
Improvement salary conditions

(B-2)

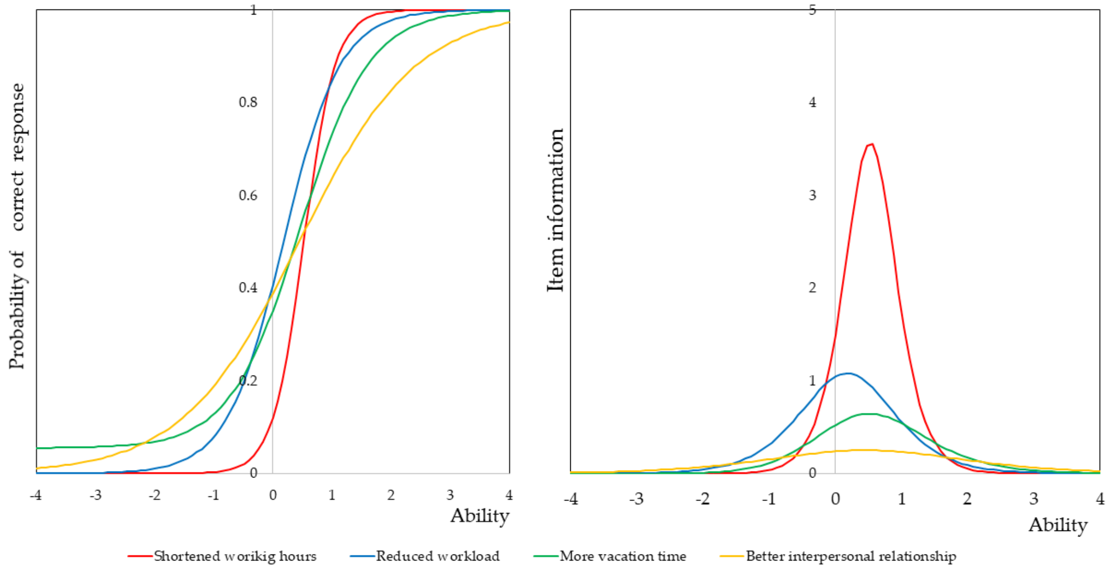
Probability of correct response
Ability
Item information
Ability
Shortened workig hours
Reduced workload
More vacation time
Better interpersonal relationship

(B-3)

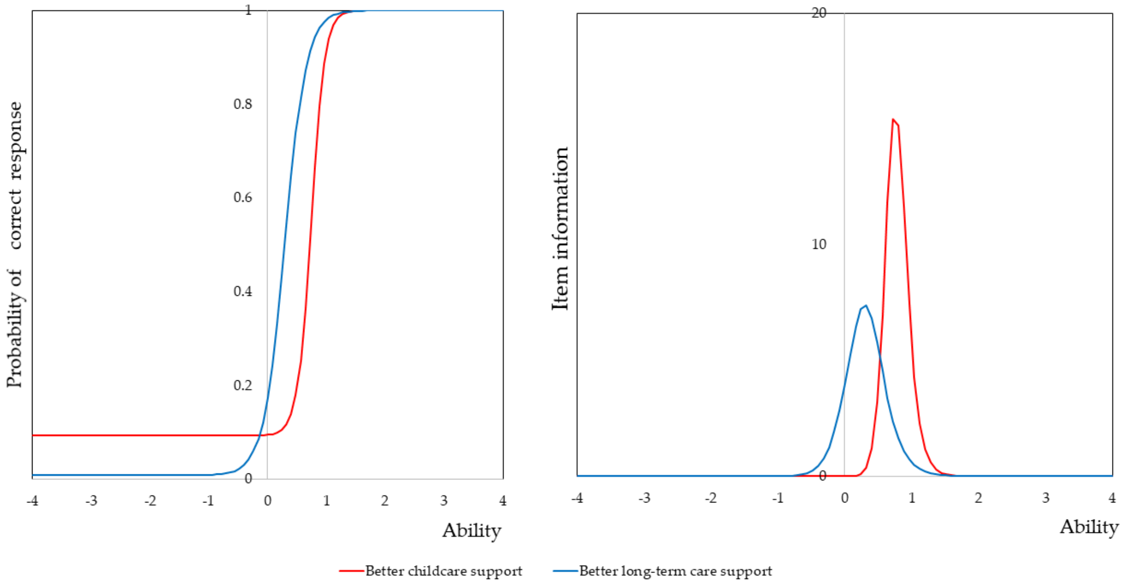

Figure S2. Item response curve and item information curve of for the items regarding job attractiveness of dental hygienists' work and the request for working environment by each factor: Job attractiveness (A), the factors dental hygienists feel would improve the work environment (B)
